# Plasma VCAM1 levels correlate with disease severity in Parkinson’s disease

**DOI:** 10.1186/s12974-019-1482-8

**Published:** 2019-05-08

**Authors:** Caroline Perner, Florian Perner, Nayana Gaur, Silke Zimmermann, Otto W. Witte, Florian H. Heidel, Julian Grosskreutz, Tino Prell

**Affiliations:** 1 El Khoury Laboratory, Center for Immunology and Inflammatory Diseases, Massachusetts General Hospital, Harvard Medical School, CNY 149-6 149 13th Street, Charlestown, MA 02129 USA; 2 Armstrong Laboratory, Department of Pediatric Oncology, Dana-Farber Cancer Institute and Harvard Medical School, 450 Brookline Ave, Boston, MA 02215 USA; 3 Institute of Clinical Chemistry and Pathobiochemistry, Otto-von-Guericke University Magdeburg, Leipziger Straße 44, 39120 Magdeburg, Germany; 4 Hans Berger Department of Neurology, Jena University Hospital, Am Klinikum 1, 07747 Jena, Germany; 5 Internal Medicine II, Hematology and Medical Oncology, Jena University Hospital, Am Klinikum 1, 07747 Jena, Germany; 6 Leibniz-Institute on Aging - Fritz Lipmann Institute, Beutenbergstraße 11, 07745 Jena, Germany; 7 Center for Healthy Ageing, Jena University Hospital, Jena, Germany

**Keywords:** Parkinson’s disease, sVCAM1, VLA4, Inflammation, Neurodegeneration, Disease severity

## Abstract

**Background:**

Parkinson’s disease (PD) is a progressive neurodegenerative disease characterized by motor and non-motor symptoms. There is increasing evidence that PD pathology is accompanied by an inflammatory response. This is highly relevant for understanding disease progression and the development of novel neuroprotective therapies.

**Objective:**

Assessing potential dysregulation of a panel of inflammatory mediators in the peripheral blood mononuclear cells (PBMCs) and plasma of PD patients and in the context of clinical outcome metrics.

**Methods:**

We performed a screening of selected cell-surface chemokine receptors and adhesion molecules in PBMCs from PD patients and age-matched healthy controls in a flow cytometry-based assay. ELISA was used to quantify VCAM1 levels in the plasma of PD patients. Lymphocytic chemotactic ability was assessed using a modified Boyden chamber assay.

**Results:**

VLA4 expression was significantly downregulated on CD3+ T cells, CD56+ NK cells, and CD3+/CD56+ NK-T cells from PD patients; further, an increase of the soluble VLA4 ligand VCAM1 in patient plasma was noted. sVCAM1 in PD patients was even higher than reported for patients with multiple sclerosis, neuromyelitis optica, and rheumatoid arthritis. sVCAM1 levels correlated with the disease stage (Hoehn and Yahr scale) and motor impairment. Chemoattraction with SDF-1α revealed impaired motility of lymphocytes from PD patients relative to controls.

**Conclusion:**

Our data provides evidence for a functional dysregulation of the sVCAM1-VLA4 axis in PD. Further studies evaluating the therapeutic potential of this axis are warranted.

**Electronic supplementary material:**

The online version of this article (10.1186/s12974-019-1482-8) contains supplementary material, which is available to authorized users.

## Introduction

Parkinson’s disease (PD) is one of the most common neurodegenerative diseases with a prevalence of 1–2 per 1000 increasing with age to 1% in the population over 60 years [1]. It is characterized by both motor and non-motor symptoms. There is increasing evidence that PD pathology is accompanied by ongoing inflammatory processess [2, 3]. This neuroinflammatory component is particularly relevant for better understanding disease progression accordingly developing disease-modifying therapies. Therefore, the present study explored dysregulated inflammatory profiles in the peripheral blood cells and plasma of PD patients within the context of established clinical indicators.

## Methods

**Fig. 1:**
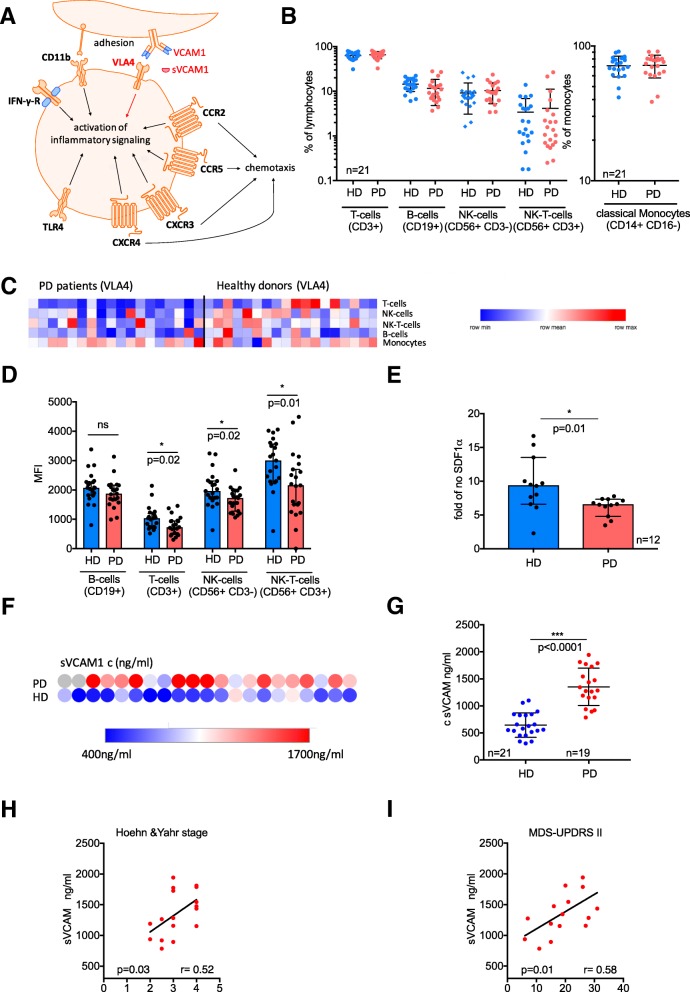
Analyses of potential alterations in key inflammatory targets in the peripheral blood mononuclear cells (PBMCs) of Parkinson’s disease (PD) patients relative to healthy donors (HDs). **a** Schematic depicting inflammatory molecules assessed by our flow cytometry-based screening assay, including chemokine receptors and adhesion molecules and their key functions. **b** Flow-cytometric subset analyses of lymphocyte and monocyte distributions in PD patients relative to HDs = displayed as percent of lymphocytes/monocytes (mean and standard deviation). **c** Heatmap of very late antigen 4 (VLA4) surface expression (mean fluorescence intensity, MFI) in PD patients and HDs on different leucocyte subsets (as measured by flow cytometry). **d** Statistical analysis of cell surface expression (MFI) of VLA4 on lymphocyte subsets from PD patients and HDs. Statistical analyses were performed using a *t*-test; ns = non-significant, *p < 0.05. **e** Boyden chamber migration assay of lymphocytes from PD patients and HDs after chemoattraction with a 100 ng/ml SDF-1α gradient. The graph shows the relative chemotactic response to SDF-1α compared to H_2_O as control. Migrated cells were counted using flow cytometry. Statistical analyses were performed using unpaired *t*-test, *p < 0.05. **f** Heatmap of sVCAM1 concentration (Invitrogen™ VCAM-1 (Soluble) Human ELISA Kit). All plasma samples from PD patients and HDs were measured in duplicate with the mean visually displayed. **g** sVCAM1 concentrations in PD patients relative to HDs. Statistical analyses were performed using the *t*-test, *p < 0.05. **h** Correlation of sVCAM1 levels observed in patients with the respective Hoehn and Yahr stages. **i** sVCAM1 concentration correlated with the MDS-UPDRS II (motor aspects of daily living) **h**, **i** Statistical analyses were performed using Pearson’s correlation, *p < 0.05

Peripheral blood mononuclear cells (PBMCs) were isolated using density gradient centrifugation from 33 PD patients (mean age 69.6 ± 10.4, 17 females) and 33 age- and sex-matched healthy donors (HDs) (mean age 63.7 ± 11.7, 13 females). Plasma samples from the same donors were retained for subsequent ELISA analysis. Multicolor flow cytometry was used to determine the expression of selected inflammatory mediators (Fig. 1a) on the surface of B cells (CD19+), T cells (CD3+), NK cells (CD56+), NK-T cells (CD56+/CD3+), and classical monocytes (CD14+/CD16−) [4]. Cellular migration was assessed using a modified Boyden chamber assay with 100 ng/ml SDF-1α as a chemoattractant [4]. Further methodological details are provided in Additional file 1.

The study was approved by the local ethics committee of the Jena University Hospital, and written informed consent was obtained from all participants. The presence of any inflammatory conditions (like diabetes, multiple sclerosis, autoimmune disease), cancer, and any current infections (as determined by clinical status, C-reactive protein (CRP), and blood leucocyte counts) constituted exclusion criteria. All PD patients were diagnosed according to the United Kingdom PD Society Brain Bank Diagnostic Criteria. Parkinson’s dementia was excluded using the Mini Mental Status Examination (MMSE). The Movement Disorder Society-sponsored revision of the Unified Parkinson’s Disease Rating Scale (MDS-UPDRS), Hoehn and Yahr staging, and the non-motor symptoms questionnaire (NMS-Quest) were used to evaluate motor and non-motor symptoms.

Data will be shared with qualified investigators upon written request.

## Results

Clinical and demographic characteristics of PD patients are provided in Table 1. The frequencies of T, B, NK, and NK-T cells and monocytes did not significantly differ between PD patients and HDs (Fig. 1b). A significant downregulation in the surface expression of the integrin “very late antigen 4” (VLA4) on T cells (*p* = 0.024), NK cells (*p* = 0.026), and NK-T cells (*p* = 0.017) was observed in PD patients (Fig. 1c, d). No alteration in TLR4, CCR2, CCR5, CXCR3, CXCR4, CD11b, and IFN-gamma-receptor expression was observed in PD patients relative to HDs (data not shown). Lymphocytes from PD patients showed diminished directed motility towards an SDF-1α gradient in a chemotaxis assay (Fig. 1e).

**Table 1:**
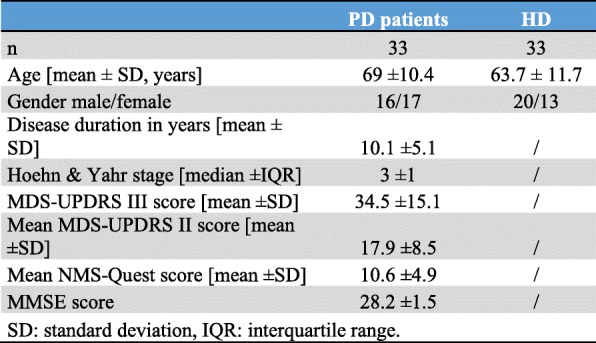
Demographic and clinical characteristics

The vascular cell adhesion protein 1 (VCAM1) is the primary ligand for VLA4. VCAM1 is expressed as a surface molecule on epithelial cell promotion and mediates lymphocyte adhesion and migration. Additionally, VCAM1 is also present as a soluble ligand circulating in the humor. Therefore, ELISA analyses of soluble VCAM1 (sVCAM1) plasma levels were performed to probe if altered lymphocytic expression of VLA4 is associated with changes in soluble ligand abundance. sVCAM1 levels were significantly elevated in PD patients relative to HDs (Fig. 1f, g). A sVCAM1 cut-off of 919 ng/ml (AUC = 0.96) had a sensitivity of 88% and specificity of 91% to discriminate between patients with PD and healthy controls (positive predictive value = 0.89, negative predictive value = 0.90, positive Likelihood ratio 9.4 [95% CI 2.5–35]). Further, sVCAM1 levels directly correlated with (a) the Hoehn and Yahr disease stage (*r* = 0.52, *p* = 0.03), (b) the MDS-UPDRS II (motor aspects of daily living; *r* = 0.58, *p* = 0.01) (Fig. 1h, i), and (c) the PDQ-39 (assessment of life quality, Additional file 1: Figure S1; *r* = 0,56, *p* = 0,04). However, no correlations were observed with age, disease duration, MDS-UPDRS III, annualized UPDRS III (data not shown), and NMS-Quest (Additional file 1: Figure S1; *r* = 0,48, *p* = 0,14). Moreover, plasma sVCAM1 levels significantly predicted individual Hoehn and Yahr stage (adjusted *R*^2^ = 0.17, *p* = 0.04).

## Discussion

The present data illustrate the role sVCAM1 levels may play in PD pathology. The levels of sVCAM1 observed here were even higher than those reported for patients with rheumatoid arthritis [5], multiple sclerosis [6], and neuro-myelitis optica [6]. Although substantial evidence exists for the association between increased sVCAM1 and age [7] and cognitive impairement [8], the use of age-matched HDs in this study has illustrated that the increase observed in PD is independent of physiological aging. Given that our study was restricted to non-demented PD patients, no correlation was observed between cognition (as assessed by the MMST) and sVCAM1 levels.

Furthermore, sVCAM1 correlated with both disease stage and the motor aspects of daily living. Both the MDS-UPDRS II and Hoehn and Yahr stage reflect long-term motor impairment in PD. This correlation might be too modest to declare sVCAM1 as a marker of Parkinson’s disease course before this effect could be proofed in larger longitudinal studies. Of note, neither of these metrics is heavily influenced by current medical treatment, unlike the MDS-UPDRS III [9]. This might potentially explain the absence of any correlation between sVCAM1 levels and the MDS-UPDRS III in the present study.

Interestingly, a recent study reported an association between sVCAM1 levels and fatigue severity in PD patients, underlining that inflammatory processes are indeed linked to distinct non-motor symptoms in PD [10].

Whether elevated sVCAM1 levels actively drive disease progression in PD or are a consequence of it remains to be fully understood. Of note, VCAM1 has already been implicated to be a potential mediator of PD pathogenesis [11]. Thus, whether targeting the VCAM1-VLA4-axis is a viable therapeutic avenue remains to be established. Indeed, promising evidence for the therapeutic potential of the VCAM1-VLA4 axis in age-related pathologies of the central nervous system already exists; the Wyss-Coray group showed that^7^ blocking VCAM1 slows down normal brain aging, induces neurogenesis, and ameliorates neuroinflammation. Our chemotaxis assay revealed diminished lymphocytic migration in PD patients which may be indicative of compromised cellular adherence and infiltration of endothelial barriers. Therefore, additional investigations and in vivo studies addressing both the expression and functional state of VCAM1 on brain endothelial cells are necessary. Future studies should also probe the relationship between VCAM1 and cognitive decline in cohorts of patients with mild cognitive impairment and PD dementia.

## Conclusion

In summary, the present study shows that plasma levels of the soluble VLA4 ligand VCAM1 are highly increased in patients with PD relative to age-matched healthy controls. Of note, sVCAM1 levels correlated significantly with disease severity (as measured by the Hoehn and Yahr scale and MDS-UPDRS II). In addition to the association with motor functionality, we observed a significant correlation with quality of life (as measured by the PDQ-39) and a trend towards a correlation with non-motor symptoms (as measured by the NMS-Quest). This data supports the hypothesis that peripheral sVCAM1 levels reflect global disease burden in patients with PD, thus indicating the potential for therapeutic targeting of the dysregulated sVCAM1-VLA4 axis.

## Additional file

Additional file 1: **Figure S1.** sVCAM1 concentration correlated with NMS-Quest (left) and PDQ-39 summary index (right). NMS-Quest measures non-motor symptoms. There is a non-significant trend to a correlation with sVCAM1 levels in PD. PDQ-39 summary index measures life-quality. There is a significant correlation of sVCAM1 with the PDQ-39 summary index. Statistical analyses were performed using Pearson’s correlation, *p < 0.05. (DOCX 78 kb)

## Abbreviations

CCR2: CC-chemokine receptor 2
CCR5: CC- Chemokine receptor 5
CD11b: Cluster of differentiation 11B
CXCR3: CXC-chemokine receptor 3
CXCR4: CXC-chemokine receptor 4
HD: Healthy donor
IFN-γ: Interferon gamma
MDS-UPDRS: Movement Disorder Society - Unified Parkinson’s Disease Rating Scale
MMSE: Mini Mental Status Examination
NMS-Quest: Non-motor symptoms questionnaire
PBMCs: Peripheral blood mononuclear cells
PD: Parkinson’s disease
PDQ-39: Parkinson’s disease questionnaire
SDF-1α: Stromal cell-derived factor 1 (CXCL12)
sVCAM1: Vascular cell adhesion protein 1
TLR4: Toll-like receptor 4
VLA4: Very late antigen-4
