# Supplementary material for: Plasma VCAM1 levels correlate with disease severity in Parkinson’s disease
Source: J Neuroinflammation. 2019 May 8;16:94. doi: 10.1186/s12974-019-1482-8 (PMC6507178; doi:10.1186/s12974-019-1482-8)
Supplement: Supplementary file 1 — Figure S1. sVCAM1 concentration correlated with NMS-Quest (left) and PDQ-39 summary index (right). NMS-Quest measures non-motor symptoms. There is a non-significant trend to a correlation with sVCAM1 levels in PD. PDQ-39 summary index measures life-quality. There is a significant correlation of sVCAM1 with the PDQ-39 summary index. Statistical analyses were performed using Pearson’s correlation, *p < 0.05. (DOCX 78 kb) [file 12974_2019_1482_MOESM1_ESM.docx]

# **Supplementary Material:**

# ****

# **Supplementary Figure 1**: sVCAM1 concentration correlated with NMS-Quest (left) and PDQ-39 summary index (right). NMS-Quest measures non-motor symptoms. There is a non-significant trend to a correlation with sVCAM1 levels in PD. PDQ-39 summary index measures life-quality. There is a significant correlation of sVCAM1 with the PDQ-39 summary index. Statistical analyses were performed using Pearson’s correlation, *p < 0.05.

## **PBMC isolation using Ficoll-Paque density gradient**

Isolation of hPBMCs from peripheral blood was perfomed using a density gradient technique with Ficoll-Paque PLUS (GE Healthcare). EDTA blood was diluted with 1× PBS/2% FBS buffer and gently layered over 15ml of Ficoll-Paque PLUS in a sterile 50ml tube. Gradients were centrifuged at 400 ×g for 25 min (brake turned off) at room temperature in a swinging rotor centrifuge. The mononuclear cell layer was carefully removed by pipetting and washed with 1xPBS/2%FBS by centrifugation at 300 x g for 10 min. PBMC pellets were resuspended in Erylysis Buffer (BD) and incubated for 3 min on ice, diluted with 1xPBS/2% FBS and washed. Pellets were then suspended in 10ml of 1 × PBS/2% FBS and cells were counted using Trypan-Blue exclusion in a hematocytometer. PBMCs were then used for subsequent assays.

## **Flow cytometry**

Surface staining of PBMCs was performed using fluorochrome-conjugated antibodies. For all flow cytometry experiments 3 million cells/tube were used from each donor; 5 tubes per donor were used in total. Lineage staining was performed with anti-CD3 (PerCP-Cy5.5), CD14 (APC-Cy7), CD19 (Brill.Violett 650), CD56 (PE), and CD16 (Brill. Violett 650) in each tube. For analysis of chemokine receptors and integrins we used anti-CXCR3 (AF488), CXCR4 (APC), CCR5 (Pe-Cy7), CCR2 (Pe-Cy7), CD11B (APC), VLA4 (Pe-Cy7) IFN-Gamma (AF488), TLR4 (PeCy7) as well as isotype controls for the respective colors. (Table 2) . Cells were stained for 30 min under light protection. Dead cell staining was perfomed via Sytox blue added to each tube shortly prior to analyses. Stained cells were measured with the BD FACSAria™ III cell sorter using the FACS DIVA (BD Biosciences) and FlowJo (LLC, USA) software packages.

**Supplementary Table 1: Antibodies used for Flow cytometry**

| **antibody** | **color** | **isotyp** | **manufacturer** | **clone** |
| --- | --- | --- | --- | --- |
| CD3 | PerCPCy5.5 | Mouse IgG, κ | Biolegend | Okt 03 |
| CD14 | APC-Cy7 | Mouse IgG, κ | Biolegend | HCD14 |
| CD19 | Brill-Violet 510 | Mouse IgG, κ | Biolegend | HIB19 |
| CD56 | PE | Mouse IgG, κ | Biolegend | HCD56 |
| CD16 | Brill. Violet 650 | Mouse IgG, κ | Biolegend | 3G8 |
| Isotyp control 1 | AF488 | Mouse IgG, k | Biolegend | MOPC-21 |
| isotyp control 2 | APC | Mouse IgG, κ | Biolegend | MOPC-21 |
| isotyp control 3 | PeCy7 | Mouse IgG, κ | Biolegend | MOPC-21 |
| Sytox blue dead cell stain | Pacific-blue | Kein Antikörper | Thermo Fischer | |
| CD11B | APC | Mouse IgG, κ | Biolegend | ICRF44 |
| VLA4 (CD49d) | PE-Cy7 | Mouse IgG, κ | Biolegend | 9F10 |
| CXCR3 | AF488 | Mouse IgG, κ | Biolegend | G025H7 |
| CXCR4 | APC | Mouse IgG, κ | Biolegend | 12G5 |
| CCR5 | PE-Cy7 | Mouse IgG, κ | Biolegend | J418F1 |
| IFN-gamma | AF488 | Mouse IgG | Novus Biologicals | 92101 |
| CCR2 | PeCy7 | Mouse IgG | Biolegend | K036C2 |
| TLR4 | PeCy7 | Mouse IgG, k | EBioscience | HTA125 |
| CD45 | APC | Mouse IgG, k | BD | HI30 |

## **Migration assay**

Post isolation, PBMCs were stimulated with 1 µg/ml Phytohaemaglutinin (PHA) and IL-2 50U/ml and incubated at 37°C for 12 hours. Cells were counted and 400000 cells were added to each chamber in 100µl RPMI with 10%FBS. In the lower chamber we added 1 µl/ml H_2_O as control or 100 ng/ml SDF1α. Cells were incubated for 2.5h at 37°C. Migrated cells to the lower compartment were counted using flow-cytometry (BD FACS CantoII). For the total cell counting and subset analysis we gated on CD45+ (APC), CD3+ cells (PerCP-Cy5.5), CD14+ (APC-Cy7), CD19+ (Brill.Violett 510) and CD56 + cells (PE).

## **Statistical analysis**

The SPSS statistical computer package (version 25.0; IBM Corporation, USA) and the GraphPad Prism (V 7.0 GraphPad Software, USA) software package were used for all statistical analyses. Values are given as the mean and standard deviation (SD) when normally distributed and otherwise as the median and interquartile range. Categorical variables are presented as numbers or percentages. Prior to statistical analysis, data were checked for outliers and for normality using the Shapiro-Wilk’s Test (*p* < 0.05). The student´s t-test was used for group comparison (corrected for multiple comparison with Bonferroni correction). Correlation between expression levels and clinical variables was tested using Pearson´s correlation for normal distribution and Spearman correlation for non-normal distributed data. Linear regression analysis was used to study the association between plasma VCAM1 levels and Hoehn & Yahr staging. Statistical significance was set at p < 0.05.

For sVCAM1 concentration the receiver-operating-characteristic (ROC) curve and the area-under-curve (AUC) were calculated to describe the ability of sVCAM1 to differentiate PD from healthy controls. Youden index was assessed to identify the optimum cut-off to distinguish PD from controls. Sensitivity, specificity, positive predictive value, negative predictive value and positive Likelihood ratio were assessed.

**Distribution of patient and healthy donor material to the respective experiments:**

Over all, 33 Parkinson´s disease patients and 33 healthy controls participated in our study. For our first approach we collected blood from 21 donors of each group for flow cytometry analysis of different surface receptors and the later ELISA assay for sVCAM1. For the sVCAM1 ELISA 21 Plasma samples of HD and 19 samples of PD were used. For the migration assay it was necessary to use freshly collected blood samples. Therefore we collected again 12 PD patients and 12 HD respectively for this approach. These patients where not identical with the individuals used for flow cytometry and ELISA before.
